# Comment on: “Quantitative Assessment of Myocardial Perfusion in Physiological and Pathological Hypertrophy Using Myocardial Contrast Echocardiography”

**DOI:** 10.1002/clc.70355

**Published:** 2026-05-21

**Authors:** Nimra Jahanzeb

**Affiliations:** ^1^ Nowshera Medical College Nowshera Pakistan; ^2^ Khyber Medical University Peshawar Pakistan

To the Editor,

We read with great interest the recent article published by Chunyao et al. evaluating myocardial perfusion differences between physiological and pathological hypertrophy using myocardial contrast echocardiography (MCE) [1]. The study addresses an important clinical challenge and provides promising evidence regarding the potential role of quantitative MCE parameters, particularly the A‐value, in differentiating an athlete's heart from hypertension‐associated hypertrophy.

However, we believe several interpretive limitations deserve further clarification.

First, the study may overattribute the observed perfusion differences to the hypertrophy phenotype itself. Coronary microvascular dysfunction in hypertensive heart disease is strongly influenced by systemic hypertension, endothelial dysfunction, vascular remodeling, and altered hemodynamics rather than myocardial hypertrophy alone. Therefore, reduced A‐values in the pathological group may reflect broader hypertension‐associated vascular dysfunction instead of a unique signature of maladaptive hypertrophy. Camici et al. previously emphasized the multifactorial nature of coronary microvascular dysfunction in hypertrophy and heart failure [2].

Second, the discussion repeatedly links the A‐value with capillary density and microvascular remodeling, although no histological validation, positron emission tomography, or cardiac magnetic resonance perfusion imaging was performed. Thus, the study demonstrates indirect perfusion differences rather than direct evidence of structural microvascular alterations [3].

Third, the diagnostic interpretation may be overstated because the ROC‐derived cutoff was developed and tested within the same small single‐center cohort without external validation. Consequently, the reported diagnostic performance may not be generalizable to broader populations [4].

Finally, the conclusions occasionally refer broadly to “pathological hypertrophy,” despite the study including only hypertension‐associated left ventricular hypertrophy. This distinction is important because other pathological hypertrophy states, such as hypertrophic cardiomyopathy or valvular hypertrophy, possess distinct microvascular pathophysiology [5].

Despite these concerns, the study provides valuable preliminary evidence supporting the role of MCE in evaluating myocardial microcirculation. We believe a clearer acknowledgment of these interpretive boundaries would strengthen the clinical and scientific implications of the findings.

## Funding

The author has nothing to report.

## Ethics Statement

The author has nothing to report.

## Consent

The author has nothing to report.

## Conflicts of Interest

The author declares no conflicts of interest.

## Data Availability

The data that support the findings of this study are available in Clinical Cardiology at https://doi.org/10.1002/clc.70285. These data were derived from the following resources available in the public domain:

## References

[1] L. Chunyao , J. Ruihan , L. Kai , et al., “Quantitative Assessment of Myocardial Perfusion in Physiological and Pathological Hypertrophy Using Myocardial Contrast Echocardiography,” Clinical Cardiology 49 (2026): e70285, 10.1002/clc.70285. 42060780 PMC13132156

[2] P. G. Camici , C. Tschöpe , M. F. Di Carli , O. Rimoldi , and S. Van Linthout , “Coronary Microvascular Dysfunction in Hypertrophy and Heart Failure,” Cardiovascular Research 116, no. 4 (2020): 806–816, 10.1093/cvr/cvaa023. 31999329

[3] K. Wei , A. R. Jayaweera , S. Firoozan , A. Linka , D. M. Skyba , and S. Kaul , “Quantification of Myocardial Blood Flow With Ultrasound‐Induced Destruction of Microbubbles Administered as a Constant Venous Infusion,” Circulation 97, no. 5 (1998): 473–483, 10.1161/01.cir.97.5.473. 9490243

[4] E. W. Steyerberg , A. J. Vickers , N. R. Cook , et al., “Assessing the Performance of Prediction Models: A Framework for Traditional and Novel Measures,” Epidemiology 21, no. 1 (2010): 128–138, 10.1097/EDE.0b013e3181c30fb2. 20010215 PMC3575184

[5] S. R. Ommen , S. Mital , M. A. Burke , et al., “2020 AHA/ACC Guideline for the Diagnosis and Treatment of Patients With Hypertrophic Cardiomyopathy: Executive Summary,” Journal of the American College of Cardiology 76, no. 25 (2020): 3022–3055, 10.1016/j.jacc.2020.08.044. 33229115

